# Regulation of contact inhibition of locomotion by Eph–ephrin signalling

**DOI:** 10.1111/jmi.12024

**Published:** 2013-03-15

**Authors:** J BATSON, JW ASTIN, CD NOBES

**Affiliations:** 1 School of Physiology and Pharmacology, University of BristolBristol, BS8 1TD, UK; 2 School of Biochemistry, University of BristolBristol, BS8 1TD, UK

**Keywords:** Cell migration, Contact inhibition of locomotion, Eph receptor

## Abstract

Contact inhibition of locomotion (CIL) occurs when a cell stops migrating in a particular direction upon contact with another cell. Many cancer cells show Contact inhibition of locomotion when contacting one another but display contact-unimpeded migration following collision with noncancer cells. Here we review current understanding of Contact inhibition of locomotion, from Abercrombie's historical studies of cells in tissue culture to more recent analyses of Contact inhibition of locomotion *in vivo*. We discuss the cellular machinery required for CIL and the molecular signals that regulate it. We focus on our recent finding that in prostate cancer cells, Contact inhibition of locomotion is regulated by a balance between EphA and EphB receptor signalling. We show that, as recently described for chick heart fibroblasts, microtubule dynamics are required for Contact inhibition of locomotion in prostate cancer cells and we propose that stabilization of microtubules could account for defective Contact inhibition of locomotion between cancer cells and noncancer cells.

## Introduction

From their observations of cell behaviour in tissue culture more than 50 years ago, Abercrombie and Heasyman noted that collisions between migrating cells *in vitro* lead to a prohibition of continued movement and a change in the direction of cell migration away from the point of cell–cell contact (Abercrombie & Heaysman, 4). They defined this contact inhibition of locomotion (CIL) as ‘the stopping of the continued locomotion of a cell in the direction that has produced a collision with another cell’ (Abercrombie, 1). By contrast, they found that many cancer cells display defective contact inhibition following collisions with noncancer cells. It was suggested that this change in migratory behaviour could facilitate cancer cell invasion, since migration away from the tumour would not be impeded and might be enhanced by interactions with stromal cells (Vesely & Weiss, 49; Abercrombie, 2). Interestingly, malignant cancer cells generally show normal CIL when contacting one another (Paddock & Dunn, 39; Astin *et al*., 7), suggesting that their failure to display CIL when contacting nonmalignant cells might be due to altered signalling rather than a general lack of contact inhibition mechanisms.

Since these historical studies of cells in tissue culture, CIL has recently also been shown to occur *in vivo* (Carmona-Fontaine *et al*., 11; Stramer *et al*., 46; Davis *et al*., 15); however, the molecular mechanisms responsible for CIL and how these are modified in contact-unimpeded migration are only starting to be uncovered and the role of CIL in cancer invasion and metastasis remains undetermined. Here we review current understanding of contact inhibition mechanisms. We discuss recent work from our laboratory, which shows that dynamic cytoskeletal rearrangements are required for CIL in heart fibroblasts and in cancer cells, and that CIL is regulated by Eph-ephrin signalling in prostate cancer cells.

## Contact inhibition of locomotion

Abercrombie & Heasyman (4) used explant confrontation experiments to investigate how cell–cell interactions influenced migratory cell behaviour. Explants of chick heart tissue were placed 1 mm apart so that fibroblast outgrowths from these explants met in the space between them. They found that when migrating cells met, they stopped moving in the direction that led to contact, and little overlap between opposing populations was observed. This contact inhibition response was studied in more detail by the use of time-lapse videomicroscopy. Using this method, Abercrombie could analyse individual cell collisions, and he found that for chick heart fibroblasts, CIL involved an initial adhesive interaction between the colliding cells, followed by a localised paralysis of the lamella and finally a contraction of the cell away from the point of contact (Abercrombie & Ambrose, 3; Abercrombie, 1).

In addition to chick heart fibroblasts, several other cell types exhibit CIL including epithelial cells, neural crest cells and haemocytes (Dunn, 16; Middleton, 35; Carmona-Fontaine *et al*., 11; Stramer *et al*., 46). When two epithelial sheets meet during wound closure they halt their membrane protrusions and stop moving forward in a contact inhibited locomotion response, although in this case the cells do not usually retract from one another and instead form adhesive interactions to fuse the sheets (Middleton, 35). The growth cones of nerve fibres also undergo pronounced cell retraction upon cell–cell contact (Dunn, 16; Kapfhammer & Raper, 27).

## CIL and malignancy

When a collision occurs between the leading edges of two fibroblasts, both cells stop moving and retract their leading edges from the point of contact (Abercrombie & Ambrose, 3; Abercrombie, 1). This is known as reciprocal CIL. However, not all cells retract from one another following cell–cell contact. For example, when the leading edge of one fibroblast contacts the side of another, the movement of the second fibroblast is not affected (Abercrombie, 1). This is called nonreciprocal CIL. Nonreciprocal CIL is often observed between normal cells and tumour cells during cell collisions. Abercrombie and Heasyman found that when the leading edge of a methylcholanthrene-induced sarcoma cell collided with a normal chick fibroblast, the migration of the sarcoma cell was unaffected by the normal cell and continued forward regardless of the obstruction (Abercrombie & Heaysman, 5). By contrast, the normal cell retracted away from the neoplastic cell and did undergo CIL. If there was nowhere for the normal cell to escape to then the sarcoma cell appeared to invade the territory of the normal cell and move under or over the normal cell (Abercrombie & Heaysman, 5). Nonreciprocal CIL was also reported for transformed rat fibroblasts colliding with normal rat fibroblasts and for human melanoma cells in collision with human skin fibroblasts, with the transformed cells failing to alter their migration upon contact with normal cells (Vesely & Weiss, 49; Stephenson *et al*., 45).

Using the explant confrontation assay, Abercrombie examined collisions between normal or malignant fibroblasts with normal chick heart fibroblasts (Abercrombie, 2). Abercrombie found that out of six tumours studied, two tumour explants showed nonreciprocal invasion into the normal fibroblast explant and that this correlated with a lack of contact inhibition in the tumour cells. Interestingly, Abercrombie and others observed that malignant cells display considerable differences in their CIL responses (Guelstein *et al*., 19; Projan & Tanneberger, 43; Vesely & Weiss, 49; Stephenson *et al*., 45). For example, Projan & Tanneberger (43) analysed CIL responses in cells from 17 normal and 29 neoplastic human tissues. They found that there was significant variation in nuclear overlap (a readout for CIL) for neoplastic cells but not normal cells. These results suggest that there may be intrinsic differences within tumour cell populations or subtypes that determine their contact inhibition responses. While many, but not all, cancer cells fail to undergo CIL towards normal cells, the same cancer cells do display CIL during collisions with one another (Vesely & Weiss, 49).

## Quantification of CIL

**Figure 1:**
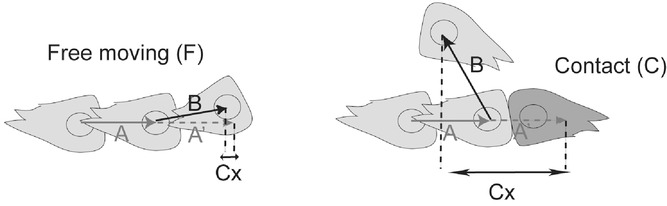
Quantification of CIL. CIL is measured by comparing the contact acceleration indices (Cx) for free moving (F) and contacting (C) cells. Cells were tracked for 50′ before collision (A) and 50′ after collision (B). Free moving cells were tracked for the same time periods. The component Cx of vector B–A represents the difference between how far the cell has progressed in the direction of A′ and how far it would have gone had there been no collision. CIL is indicated by a negative Cx value because cells change direction and move backwards following the collision. A more negative Cx value indicates a more significant CIL response.

In their early studies on cell locomotion, Abercrombie and Heasyman fixed their explant cultures after the outgrowths had met and measured the degree of nuclear overlap as an indication of contact inhibition (Abercrombie & Heaysman, 4). Several other investigations using this method were subsequently reported (Projan & Tanneberger, 43; Stephenson *et al*., 45). However, this method is misleading when colliding cells do not respond equally to the collision as observed during nonreciprocal collisions. Instead, individual collisions were analysed, first qualitatively (Abercrombie & Ambrose, 3) and then quantitatively (Guelstein *et al*., 19; Vesely & Weiss, 49; Paddock & Dunn, 39; Carmona-Fontaine *et al*., 11; Astin *et al*., 7). Paddock and Dunn applied a vector analysis to analyse collisions between cells. The displacement of a migrating cell for a period of time before collision (Fig. 1, vector A) and for the same period of time following collision (Fig. 1, vector B) is measured. The contact acceleration index (Cx) of vector *B*–*A* represents the difference between how far the cell has progressed and how far it would have gone had there been no collision (Fig. 1, vector A′). Cx values were also calculated for the same population of cells that were free-moving and not colliding over the same time frames. CIL was considered to have occurred when the mean Cx value of colliding cells (C) was significantly different to that of free-moving cells (F) as measured by Mann–Whitney statistical tests. Cx measurements were scaled to ignore differences in speed between cell populations. This method of quantification is useful for determining whether cells undergo the whole process of CIL, from initial contact and arrest of cell migration to retraction, repolarization and reinitiation of migration in a new direction. Each of these steps could be investigated in more detail using further quantification methods such as the length of contact time between colliding cells or analysis of centrosome, Golgi or cytoskeletal reorientation following cell–cell contact.

## Molecular mechanisms of CIL

Upon contact, cells stop migrating, retract their actin-driven protrusions, repolarize and form a new protrusion to reinitiate migration in a new direction. The molecular signals required for each of these steps are largely unknown but some progress has been made in recent years.

Work from the Mayor laboratory has shown that the PCP (noncanonical) Wnt pathway is involved in CIL in Xenopus neural crest cells (Carmona-Fontaine *et al*., 11; Matthews *et al*., 34). These studies showed that inhibition of noncanonical Wnt signalling inhibited both CIL and the directionality of neural crest migration. Wnt-signalling members were localized at the site of cell contact, leading to the activation of RhoA at this site. Activation of RhoA at the cell contact could lead to the collapse of membrane protrusions and a change in cell polarity, thereby directing migration away from the cell contact. Carmona-Fontaine *et al*., suggest that CIL could be sufficient for the directional migration of neural crest cells *in vivo* and may direct the migration of groups of cells during development (Carmona-Fontaine *et al*., 11). The importance of CIL for embryo patterning and morphogenesis during development has been confirmed recently by the Stramer laboratory, who have used mathematical modelling to show that CIL in haemocytes in the Drosophila embryo can explain the final pattern of haemocyte distribution (Davis *et al*., 15).

Recent work in our laboratory showed that RhoA/ROCK signalling is required for CIL in chick heart fibroblasts (Kadir *et al*., 26). Inhibition of ROCK led to a loss of cell–cell repulsion and a failure of CIL. RhoA and ROCK are known to mediate actin contractility; however, these cells exhibited almost normal CIL properties following inhibition of myosin contractility by treatment with blebbistatin. Instead, it was found that the RhoA/ROCK pathway mediates CIL by regulating the microtubule cytoskeleton (Kadir *et al*., 26). During collisions, microtubules at points of cell–cell contact increase their frequency of catastrophe and their rates of shrinkage and growth. Dynamic reorganization of the microtubule network was required for cells to switch their front-rear polarity to undergo CIL. In ROCK-inhibited cells, microtubules were stabilized leading to failure of CIL, which could be rescued by microtubule destabilization using nocodazole treatment (Kadir *et al*., 26). These results suggest that the switch in cell polarity away from the point of contact is a key driver in the CIL response. Dynamic microtubules have also been shown to be important mediators of cell polarity and contact inhibition responses in Drosophila macrophages *in vivo* (Stramer *et al*., 46).

Other studies have found that integrins and cadherins may also be involved in CIL (Chen & Obrink, 12; Bracke *et al*., 10; Huttenlocher *et al*., 24; Ayollo *et al*., 8; Theveneau *et al*., 48). Inhibition of N-cadherin using a blocking antibody in neural crest cells led to failure of CIL. Knockdown of N-cadherin using morpholinos resulted in failure of neural crest cells to repolarize upon contact with other cells and instead these cells formed protrusions on top of one another. N-cadherin was found to colocalize at cell–cell contacts with p120-catenin and beta-catenin, where it acted to downregulate Rac1 activity and thereby prevent the formation of cell protrusions between the cells (Theveneau *et al*., 48).

The Rho GTPases are key regulators of the actin cytoskeleton and cell polarity and are likely to play important roles during CIL (Carmona-Fontaine *et al*., 11; Astin *et al*., 7; Theveneau *et al*., 48; Anear & Parish, 6). Anear & Parish (6) used Abercrombie's explant confrontation assay and showed that dominant active Rac1, dominant negative Rac1 or an increase in RhoA activity led to loss of contact inhibition in NIH3T3 cells confronting chick heart fibroblasts. Another recent study showed that Nm23-H1 is required for CIL in glioma cells by suppressing the Rac guanine nucleotide exchange factor (GEF) Tiam1 at sites of cell–cell contact (Tanaka *et al*., 47). In addition ephrin-B1 was found to inhibit the association of Nm23-H1 with Tiam1, leading to failure of CIL and increased cell invasion (Tanaka *et al*., 47).

Eph receptors and their ephrin ligands are good candidates for regulators of CIL because they are both membrane tethered and activated by cell–cell contact and their downstream signalling can control cell migration via regulation of the actin cytoskeleton. Recent work by our laboratory has shown that CIL can be regulated by the interaction between Eph receptors and their ephrin ligands at cell–cell contact sites (Astin *et al*., 7). Our data have shown that the ability of a cell to undergo CIL upon cell–cell collision depends on a balance between signalling from the two classes of Eph receptor, EphA and EphB (Astin *et al*., 7).

## Eph–ephrin signalling

Eph receptors are the largest subfamily of receptor tyrosine kinases. In the human genome there are nine EphA receptors, which bind to five glysosylphosphatidylinositol-linked ephrin-A ligands, and five EphB receptors, which bind transmembrane ephrin-B ligands (Klein, 28). Generally EphA receptors bind ephrin-A ligands and EphB receptors bind ephrin-B ligands, although there are some exceptions to these rules (Himanen *et al*., 22; Pasquale, 42). Eph receptor–ephrin ligand interactions depend upon direct cell–cell interactions and are unique in that they trigger bidirectional signalling within the receptor and ligand-expressing cell. Upon binding their ephrin ligands, Eph receptors cluster together and heterodimerize, leading to receptor autophosphorylation on several intracellular tyrosine residues (Himanen *et al*., 22). Ephrin-B ligands can also be phosphorylated on their intracellular domains.

Eph receptors and their ephrin ligands have well described roles in vascular development, tissue boundary formation, and axon guidance (Kullander & Klein, 30; Pasquale, 42). The best described outcome of Eph–ephrin signalling is a repulsive response involving cell retraction, for example during axon guidance or cell–cell compartmentalization events during development or in the adult intestine (Batlle *et al*., 9; Egea & Klein, 17). In the intestinal epithelium, restricted expression of Eph receptors and ephrins prevents intermingling of cell populations and thereby controls the positioning of cell types along the crypt-villus axis (Batlle *et al*., 9). Alternatively, adhesive and attractive responses can occur following cell–cell interactions (Holmberg *et al*., 23; Pasquale, 41). The mechanisms that mediate repulsive versus attractive migration are not completely understood. It has been suggested that the level of signalling may be important, such that low levels of Eph forward signalling can mediate attraction responses that switch to repulsion when signalling intensity increases (Pasquale, 41). Another possibility is that the degree of Eph receptor clustering might change cellular outcome or that opposing responses can occur at different points in time, so that an initial response might be attractive but that this might then switch to repulsion. A different hypothesis, supported by our recent data, is that a combinatorial code of specific Eph receptor–ephrin interactions dictates whether a cell will respond with repulsive or attractive migration (Astin *et al*., 7).

Eph–ephrin interactions regulate cell morphology, adhesion and migration by signalling to the actin cytoskeleton, particularly via their effects on the small Rho GTPases. Generally, activation of EphA receptors leads to repulsive migratory behaviour via the GTPase RhoA (Wahl *et al*., 50; Shamah *et al*., 44; Lawrenson *et al*., 32; Ogita *et al*., 38; Astin *et al*., 7) whereas EphBs can trigger Cdc42 activation and this can facilitate attractive migration (Irie & Yamaguchi, 25; Astin *et al*., 7).

## Contact inhibition responses between prostate cancer cells and stromal cells

To investigate the mechanisms underpinning CIL and how cancer cells are able to overcome them during collisions with noncancerous cells, we used classic CIL experiments with prostate cancer cell lines and stromal fibroblasts *in vitro*. PC-3 and DU-145 are two prostate cancer cell lines that are motile in tissue culture in the presence of hepatocyte growth factor (HGF) (Astin *et al*., 7). PC-3 and DU-145 are both tumourigenic, but only PC-3 cells are efficient at forming metastases when injected subcutaneously into mice (Kozlowski *et al*., 29). PC-3 cells are more invasive than DU-145 cells in transwell invasion assays *in vitro* (Astin *et al*., 7).

**Figure 2:**
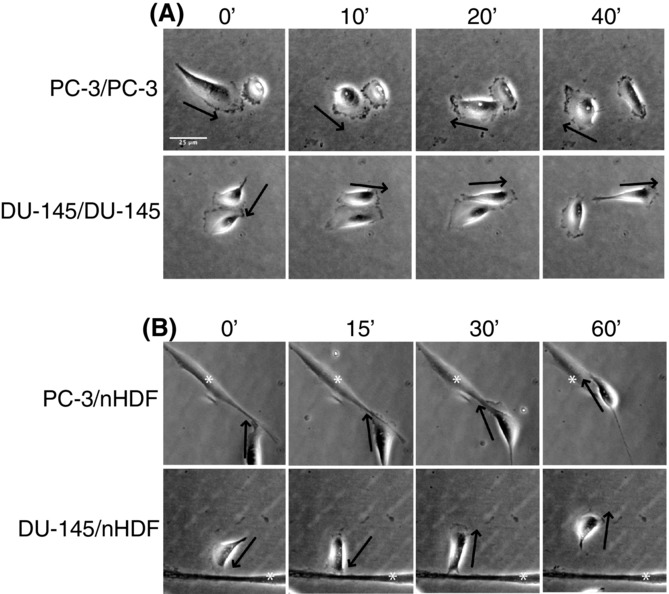
Contact inhibition responses between prostate cancer cells and between prostate cancer cells and stromal cells. Representative images from time-lapse movies at the indicated timepoints from collisions between two prostate cancer cells; PC-3/PC-3 (Supporting Movie S1) and DU-145/DU-145 (Supporting Movie S2) (A) and between prostate cancer cells and fibroblasts; DU-145/nHDF (Supporting Movie S4) and PC-3/nHDF (Supporting Movie S3) (B) Arrows indicate the direction of cell migration of the cancer cell and asterisks indicate fibroblasts (nHDF).

When PC-3 or DU-145 prostate cancer cells collide with another cell of the same type, both cells undergo contact inhibition much like the chick heart fibroblasts initially studied by Abercrombie (Fig. 2A; Supporting Movies S1 and S2). The cancer cells stop moving in the direction that brought them into contact, retract their membrane protrusions, repolarize and form a new lamellipodia to reinitiate migration away from the collision. In collisions between DU-145 cells and normal fibroblasts (nHDF, normal human dermal fibroblasts), the DU-145 cell displays the same CIL response and is repelled by the fibroblast (Fig. 2B, Supporting Movie S4). By contrast, during collisions between PC-3 cells and fibroblasts, the PC-3 cell fails to undergo CIL and instead displays defective CIL (Fig. 2B, Supporting Movie S3). PC-3 cells can protrude underneath the fibroblast and often seem attracted to the fibroblast, despite attempts by the fibroblast to retract away from the collision.

## EphA receptors mediate CIL between prostate cancer cells

**Figure 3:**
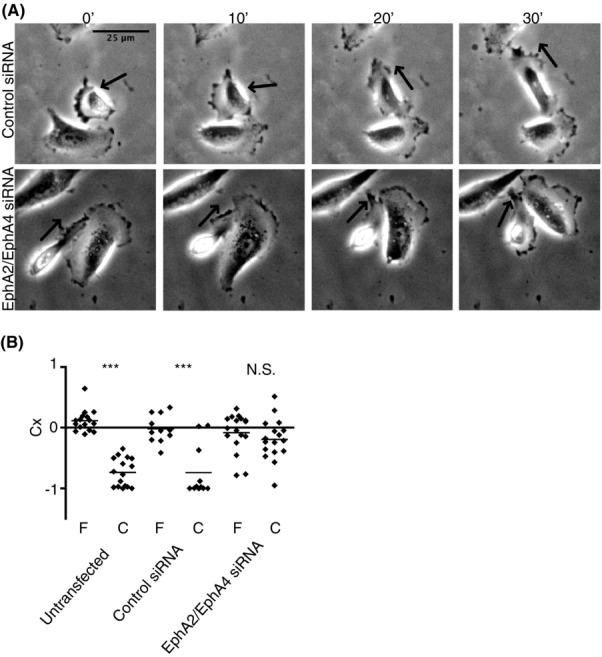
EphA receptors mediate CIL between prostate cancer cells. Representative images from time-lapse movie of PC-3 cells treated with 25 nM control siRNA (Supporting movie S5) or EphA2 + EphA4 siRNA (Supporting Movie S6) (A) (see Astin et al., 7 for details of oligonucleotides). Contact acceleration indices (Cx) of free moving (F) and colliding (C) cells for untransfected cells (n = 16), control siRNA-treated cells (n = 11) or EphA2+EphA4 siRNA-treated cells (n = 17). Triple asterisks indicate p < 0.001, N.S. not significant, determined by a Mann–Whitney test. Data are from at least three independent experiments.

Eph receptors and their ephrin ligands are good candidates for mediators of CIL because they are both membrane bound therefore activated upon cell–cell contact and have well recognized roles in repulsive migratory responses. We previously showed that ephrin-A ligands are sufficient to induce CIL responses in PC-3 cells (Astin *et al*., 7). In PC-3 cells, we know that CIL is mediated by EphA2 and EphA4 (Fig. 3A, Supporting Movies S5 and S6 (Astin *et al*., 7) because knockdown of these two EphA receptors led to an inhibition of the cell repulsion response. Instead, PC-3 cells carried on moving in the same direction regardless of cell–cell contact and pushed past each other. We quantified CIL based on the method established by Paddock and Dunn (39). Cells were tracked before and after individual collisions and during a period of free movement and vector analysis was used to measure how cell migration deviated from a straight line following contact. The Cx value represents this deviation. A significant difference is seen between Cx values for free moving and colliding PC-3 cells during PC-3:PC-3 collisions (*p* < 0.001, Mann–Whitney test). This indicates that CIL has occurred. However, when PC-3 cells are treated with EphA2/EphA4 siRNA, the difference between free migration and migration following contact was significantly reduced, indicating that these cells do not display CIL (Fig. 3B, not significant (N.S.) Mann–Whitney test, Supporting Movies S5 and S6 Astin *et al*., 7). These data are consistent with previously described roles of EphA receptors in repulsive cell responses in the nervous system where they mediate growth cone collapse (Shamah *et al*., 44).

## Microtubule dynamics are required for the switch in cell polarity during CIL

**Figure 4:**
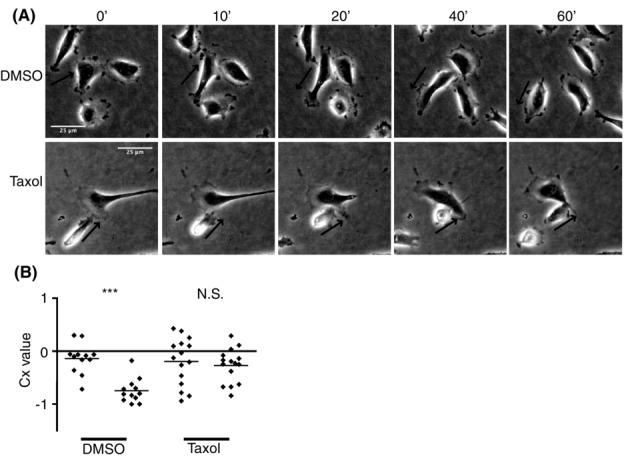
Microtubule dynamics are required for the switch in polarity during CIL. Representative images from time-lapse movies of cell–cell collisions between PC-3 cells pretreated with DMSO (Supporting Movie S7; n = 12) or Taxol (5 nM) (Supporting Movie S8; n = 15) for 5 h (A). Contact acceleration indices (Cx) of free moving (F) and colliding (C) cells. Arrows indicate the direction of cell migration. Data are from at least three independent experiments.

Work in our laboratory has shown that at sites of cell–cell contact during cell collisions, microtubules exhibit increased catastrophe frequency and increased rates of shrinkage and growth (Kadir *et al*., 26). In chick heart fibroblasts, the switch in polarity mediated by microtubule reorganization and formation of a new leading edge away from the point of contact are key events during CIL. Here we show that in PC-3 cells, treatment with low concentrations of taxol (5 nM) to stabilize microtubules, without inhibiting cell migration, also leads to failure of CIL (Fig. 4, Supporting Movies S7 and S8). Taxol-treated cells do not display the switch in cell polarity seen in DMSO control-treated cells and instead slide past each other or even protrude beneath each other (Fig. 4). The Cx values of free-moving and contacting DMSO-treated cells are significantly different indicating that CIL has occurred (*p* < 0.001, Mann–Whitney test). There is no significant difference between the free and contact Cx values of taxol-treated cells indicating that taxol treatment leads to failure of CIL (Fig. 4, N.S. Mann–Whitney test).

## EphB receptors mediate contact-unimpeded migration during collisions between PC-3 cells and fibroblasts

**Figure 5:**
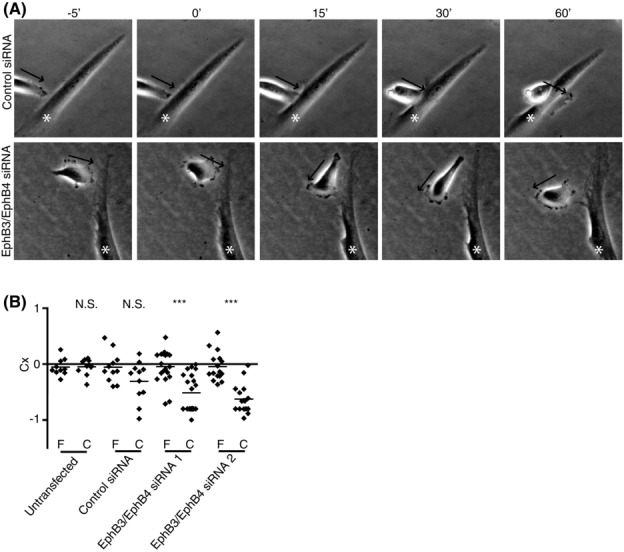
EphB receptors mediate contact-unimpeded migration during collisions between PC-3 cells and fibroblasts. Representative images from time-lapse movies of PC-3 cells treated with control (Supporting Movie S9) or EphB3+EphB4 siRNA (Supporting Movie S10) (A) (see Astin et al., 7 for details of oligonucleotides). Contact acceleration indices of free moving (F) and colliding cells (C) for untransfected cells (n of 13), control siRNA-treated cells (n = 11) or EphB3+EphB4 siRNA-treated cells (siRNA-oligonulceotide 1; n = 30, oligonucleotide 2; n = 16). Triple asterisks indicate p < 0.001, N.S., not significant, determined by a Mann–Whitney test. Arrows indicate the direction of cancer cell migration, asterisks indicate fibroblasts (nHDF). Data are from at least three independent experiments.

Reverse-transcription PCR profiling of the Eph receptor and ephrin expression in PC-3 and DU-145 cells indicated that PC-3 cells have increased expression of EphB3 and EphB4 compared to DU-145 cells (Astin *et al*., 7). Furthermore, the predominant ligand for EphB4, ephrin-B2 enhanced PC-3 cell migration in transwell migration assays and was expressed at higher levels by fibroblasts (Astin *et al*., 7). We therefore asked whether defective CIL in PC-3 cells colliding with fibroblasts might be mediated by EphB–ephrinB signalling. Untransfected PC-3 cells and PC-3 cells treated with control siRNA have contact-unimpeded migration during collisions with fibroblasts (Fig. 2B, Supporting Movie S3, Fig. 5A, Supporting Movie S9). This results in no significant difference between free movement and movement following collision in these cells (Fig. 5B N.S. Mann–Whitney). However, when EphB3 and EphB4 are knocked down in PC-3 cells treated with siRNAs, CIL is restored (Fig. 5B, Supporting Movie S10 Astin *et al*., 7). These prostate cancer cells do not keep moving in a continued direction past or underneath the contacted fibroblast but instead retract and repel away after collision. This is indicated by a significant difference in Cx values (Fig. 5B *p* < 0.001 Mann–Whitney test) and suggests that defective CIL in heterotypic collisions between PC-3 cells and fibroblasts is mediated by EphB3 and EphB4 signalling. DU-145 cells may not display defective CIL because they do not have increased expression of EphB receptors and so EphA signalling predominates and CIL occurs in heterotypic collisions between DU-145 cells and fibroblasts (Astin *et al*., 7).

## Discussion

Here we show that CIL between prostate cancer cells is regulated by EphA receptors, specifically EphA2 and EphA4. These receptors appear to act together to coordinate CIL. By contrast, PC-3 cells display contact-unimpeded migration following collisions with fibroblasts. We find that this is due to their increased expression levels of EphB3 and EphB4, which engage ephrin-B2 expressed on fibroblasts. Knockdown of these two EphB receptors can restore CIL between PC-3 cells and fibroblasts. We propose that during cell–cell collisions, cell migratory responses are regulated by a balance between repulsive EphA versus attractive EphB signalling.

Recently, we have shown that Cdc42 is activated downstream of EphB receptors while RhoA is activated following EphA receptor activation (Astin *et al*., 7). We hypothesize that these RhoGTPases mediate CIL responses downstream of Eph receptor activation via their effects on the cytoskeleton. This is consistent with studies in other systems. For example, in hippocampal neurons, stimulation with soluble ephrin-B2 leads to recruitment of the Cdc42 GEF, intersectin, to EphB2 and activation of this GEF. This leads to activation of Cdc42, which mediates spine morphogenesis (Irie & Yamaguchi, 25). By contrast, activation of RhoA promotes growth cone collapse. RhoA signalling mediates contractility and Rac has been shown to control Eph receptor endocytosis, both of which are required for repulsive migration (Marston *et al*., 33). Activation of Rho and Rac occurs by Eph receptor recruitment and activation of the GEFs, ephexin1 and Vav2, (Shamah *et al*., 44; Cowan *et al*., 13). EphA receptors mediate PC-3 cell retraction via a signalling pathway involving RhoA, ROCK and leading to actomyosin contractility (Astin *et al*., 7). This may be important for retraction of cell protrusions upon cell–cell contact during CIL. It is currently unknown whether Eph signalling influences cell repolarization following cell–cell contact. Recent work has shown that rearrangement of the microtubule cytoskeleton is important for the front-rear switch in polarity required for cells to migrate in a new direction away from points of cell–cell contact. In chick heart fibroblasts ROCK inhibition leads to failure of CIL due to stabilization of microtubules, suggesting that RhoA-ROCK signalling may destabilize microtubules during CIL. Partial destabilization of microtubules restored CIL in ROCK-inhibited cells (Kadir *et al*., 26). Previous studies have shown that stable microtubules are depleted from points of cell–cell contact (Gundersen & Bulinski, 21; Nagasaki *et al*., 36) and recent work from our laboratory has shown that microtubule dynamics are increased at points of contact during cell collisions (Kadir *et al*., 26). Here we show that stabilization of microtubules using low doses of taxol leads to failure of CIL in PC-3 cells. Nanomolar concentrations of taxol have previously been shown to increase the proportion of stable microtubules (Kadir *et al*., 26). We hypothesize that microtubule stabilization might account for EphB-mediated defective CIL between PC-3 cells and fibroblasts. Microtubules are known to assemble into a polarized array in migrating cells and stable microtubules extend to the leading edge in the direction of migration (Wittmann & Waterman-Storer, 53; Watanabe *et al*., 51; Stramer *et al*., 46). Several studies have shown that microtubule stabilization promotes directionally persistent migration (Gundersen, 20; Wen *et al*., 52; Watanabe *et al*., 51). We have shown that Cdc42 is activated downstream of EphB receptors and is functionally required for attractive migration towards ephrin-B2 in transwell migration assays (Astin *et al*., 7). Cdc42 is a key mediator of cell polarity and may regulate microtubule stabilization via several mechanisms. For example, Cdc42 could promote microtubule capture at the plasma membrane by binding and recruiting IQGAP1 leading to interaction with the +TIP CLIP170 (Fukata *et al*., 18). Alternatively, Cdc42 could regulate the stability of microtubules through activation of PAK, which can phosphorylate and inhibit the microtubule destabilizing protein stathmin (Daub *et al*., 14). Further work will test the role of microtubules in CIL and in contact-unimpeded migration during collisions between PC-3 cells and fibroblasts and how this is regulated by Eph receptor-Rho GTPase signalling.

Our finding that EphB4 is increased in the more metastatic cell line, PC-3, compared to the less metastatic prostate cancer cell line, DU-145, is consistent with previous reports (Xia *et al*., 54). Indeed, Eph receptor expression is upregulated in many malignancies including breast, prostate and colon (Xia *et al*., 54; Kumar *et al*., 31). We, and others, have found that EphB4 is expressed in advanced but not in benign prostate tumour tissues (Xia *et al*., 54; Noren & Pasquale, 37; Astin *et al*., 7). Although the role of Eph receptors in cancer progression is complex, and EphB4 has been shown to have tumour promoting and inhibitory functions, EphB4 is generally thought to convey a more invasive phenotype. This is consistent with the hypothesis first put by Abercrombie, that contact-unimpeded migration could facilitate tumour invasion through the stroma. We hypothesize that upregulation of EphB4 expression in metastatic prostate cancer cells could enhance their invasion through deregulation of contact inhibition. Migration away from the primary tumour could be facilitated if repulsive contact inhibition restraints are overcome and prostate cancer cells can keep moving through the territory of the stromal cells without hindrance. Ongoing work will investigate this hypothesis.

Since Abercrombie's early observations on the social behaviour of migrating cells in tissue culture, several studies have shown that CIL is an important process in defining the migratory behaviour of cells *in vitro* and *in vivo* (Abercrombie & Heaysman, 4; Abercrombie, 1; Carmona-Fontaine *et al*., 11; Astin *et al*., 7; Theveneau *et al*., 48). We now have an understanding of the molecules involved in CIL and how defective CIL is mediated in cancer cells contacting noncancer cells. This work will enable further investigation of the signalling mechanisms underlying CIL responses and their functional outcomes, particularly in the investigation of the role of CIL in cancer cell invasion.

## Supporting Information

Movie S1 Phase time-lapse images of two PC-3 cells colliding. Frames taken every 15 s and displayed at 12 fps.

Movie S2 Phase time-lapse images of two DU-145 cells colliding. Frames taken every 15 s and displayed at 12 fps.

Movie S3 Phase time-lapse images a PC-3 cell colliding with a fibroblast. Frames taken every 15 s and displayed at 12 fps.

Movie S4 Phase time-lapse images a DU-145 cell colliding with a fibroblast. Frames taken every 15 s and displayed at 12 fps.

Movie S5 Phase time-lapse images of collisions between PC-3 cells treated with control siRNA. Frames taken every 15 s and displayed at 12 fps.

Movie S6 Phase time-lapse images of collisions between PC-3 cells treated with EphA2 and EphA4 siRNA. Frames taken every 15 s and displayed at 12 fps.

Movie S7 Phase time-lapse images of collisions between PC-3 cells treated with DMSO. Frames taken every 15 s and displayed at 12 fps.

Movie S8 Phase time-lapse images of collisions between PC-3 cells treated with 5 nM taxol. Frames taken every 15 s and displayed at 12 fps.

Movie S9 Phase time-lapse images of a PC-3 cell treated with control siRNA colliding with a fibroblast. Frames taken every 15 s and displayed at 12 fps.

Movie S10 Phase time-lapse images a PC-3 treated with EphB3 and EphB4 siRNA colliding with a fibroblast. Frames taken every 15 s and displayed at 12 fps.
